# Methylene Blue-Enriched Feed Mitigates Acute Nitrite Intoxication in Nile tilapia (*Oreochromis niloticus*)

**DOI:** 10.3390/ani16071042

**Published:** 2026-03-29

**Authors:** Karoline M. Barbuio, Gustavo H. G. Pinto, Brunno S. Cerozi

**Affiliations:** Department of Animal Science, College of Agriculture, University of Sao Paulo, Avenida Padua Dias, 11, Piracicaba 13418–900, SP, Brazil; karolinembarbuio@usp.br (K.M.B.);

**Keywords:** methylene blue, medicated feed, RAS, nitrite poisoning, methemoglobinemia

## Abstract

Intensive recirculating aquaculture systems are vulnerable to spikes of nitrite, which oxidizes hemoglobin to methemoglobin and compromises oxygen transport. Methylene blue (MB) is a classical antidote for methemoglobinemia, yet its use in fish has been limited to injections or immersion baths that are impractical for large-scale operations. This study assessed whether MB incorporated into a medicated feed could mitigate acute nitrite intoxication in Nile tilapia. Fish received either a control diet or 0.1% MB diet. After five days on the experimental diets, fish were exposed to nitrite for 48 h. Control fish experienced five deaths, whereas no mortality or behavioral distress was observed in MB-treated fish. Hematology indicated significantly lower circulating methemoglobin concentration in the MB group, while the control fish had higher hemoglobin concentration and erythrocyte counts, consistent with compensatory erythropoiesis. Gill histology revealed preserved lamellae with only mild changes in MB-fed fish, whereas control fish displayed lamellar aneurysm, edema, capillary congestion, fusion and epithelial hyperplasia. Therefore, oral MB administration appears to ameliorate the physiological consequences of acute nitrite exposure, offering a scalable intervention for emergency management of nitrite spikes. Future work should define dose–response relationships, evaluate post-exposure rescue, quantify gill lesions and assess MB residues in food fish.

## Simple Summary

Nitrite is a harmful form of nitrogen that can quickly build up in fish farms when filtration systems fail. Nitrite causes damage mostly by turning hemoglobin into methemoglobin, which cannot carry oxygen. As a result, fish may suffer from oxygen deprivation, stress and even death. We tested whether adding a small amount of methylene blue (MB), a redox dye used in medicine, to the feed of Nile tilapia could protect them during an acute nitrite crisis. Fish fed the MB diet for five days were exposed to high levels of nitrite. None of these fish died or showed signs of distress, while several fish on a normal diet died and showed sluggish behavior. Blood tests showed that MB-fed fish had lower levels of oxidized hemoglobin and did not need to boost their red blood cell counts as much as untreated fish. Their gills also looked healthier with fewer lesions. These results suggest that MB can be delivered through feed to reduce the harmful effects of nitrite poisoning and improve fish welfare during emergencies. However, further studies are needed to confirm safe doses, treatment duration and any potential residues in edible tissues.

## 1. Introduction

Recirculating aquaculture systems (RASs) maximize production while minimizing water use, but they are susceptible to rapid accumulation of nitrogenous metabolites such as nitrite [1]. Nitrite is an intermediate of nitrification that, once absorbed across the gills, diffuses into erythrocytes and oxidizes hemoglobin iron from the ferrous (Fe^2+^) to ferric (Fe^3+^) state, producing methemoglobin [2]. Methemoglobin cannot carry oxygen, so rising methemoglobin fractions progressively impair oxygen delivery and lead to tissue hypoxia [3]. Beyond impaired oxygen transport, nitrite intoxication induces metabolic, cardiovascular, and immunological disturbances and can trigger histopathological lesions in multiple organs [4].

Routine countermeasures against nitrite spikes include adding chloride salts to compete with nitrite uptake by the gills, temporarily reducing feeding, and performing partial water exchange to dilute nitrite concentrations [5]. These approaches may delay nitrite entry in the bloodstream but do not reverse methemoglobinemia once it develops, and they can be problematic in recirculating systems (e.g., sodium chloride build-up) due to their negative impact on biofilter performance [6]. Methylene blue (MB) is a redox-active dye that is widely used to treat methemoglobinemia in humans and other animals by acting as an electron carrier that reduces methemoglobin back to functional hemoglobin [7]. In aquaculture, MB has been applied as an antimicrobial and disinfectant [8,9], and limited studies have tested it for nitrite intoxication using intraperitoneal injection [10] or immersion bath [11].

While methylene blue has traditionally been administered after intoxication through injection or immersion baths, these approaches are labor-intensive and difficult to deploy during acute water quality crises in large aquaculture systems. An alternative strategy would be to maintain a circulating redox mediator capable of buffering hemoglobin oxidation during nitrite exposure. If such protection could be achieved through routine feeding, it would provide a practical method to physiologically stabilize fish during transient nitrite spikes without handling or chemical treatment of system water. To our knowledge, no study has evaluated whether methylene blue delivered orally through medicated feed can attenuate nitrite-induced methemoglobinemia in fish. A feed-based strategy could be deployed immediately during routine feeding, avoiding handling stress and minimizing chemical use in recirculating systems. Therefore, the objective of this study was to evaluate the effectiveness of a 0.1% MB inclusion in feed for reducing methemoglobin burden, improving functional oxygen transport, and mitigating mortality and tissue injury during an acute nitrite challenge in Nile tilapia.

## 2. Materials and Methods

### 2.1. Ethical Approval and Study Site

All procedures involving animals were approved by the Ethics Committee on Animal Use of the University of São Paulo (protocol no. 2036290823). Experiments were conducted at the Fish Nutrition Laboratory of the College of Agriculture (Esalq/USP) in Piracicaba, Sao Paulo, Brazil.

### 2.2. Experimental Animals and Husbandry

Sixty juvenile Nile tilapia (*Oreochromis niloticus*) with an initial mass of 25.0 ± 1.3 g were randomly assigned to four glass tanks (70 L; 15 fish per tank). Two tanks per treatment were maintained as redundant husbandry units to minimize the risk of total stock loss in case of system failure; however, since tanks within each treatment received the same water source, feeding protocol, and environmental conditions, they functioned as parallel husbandry units rather than independent experimental treatments. Therefore, individual fish were considered the appropriate observational units for physiological measurements. Tanks were supplied with continuous flow-through water and constant aeration via air stones. Fish were acclimated for seven days on the basal diet before the trial. Water quality parameters were monitored daily using a portable probe (Horiba U-50 multiparameter probe, Kyoto, Japan) and colorimetric kits (API Ammonia Test Kit, Chalfont, PA, USA) to ensure that conditions remained within ranges suitable for tilapia culture (dissolved oxygen 6.8 ± 0.3 mg/L, temperature 27.5 ± 0.8 °C, pH 6.98 ± 0.10, total ammonia 1.25 ± 0.43 mg/L). and did not confound the effects of nitrite exposure.

### 2.3. Diet Formulation and Medicated Feed Preparation

A basal diet was formulated to meet the nutritional requirements of juvenile tilapia, providing 26% crude protein and 3200 kcal/kg digestible energy (Table 1).

Ingredients were ground, mixed, moistened and extruded at 120 °C. After drying, the pellets were ground again and divided into two equal portions, in which one half was supplemented with 10 g/kg of reagent-grade methylene blue (resulting in a 0.1% inclusion), mixed, re-pelleted and dried. The other half was re-pelleted without any supplement. Both diets were stored at 4 °C until use. Fish were fed the control feed to apparent satiation three times daily (08:00, 12:00 and 16:00) for seven days. Following the seven-day acclimation period, the MB group received the medicated feed for five days prior to nitrite exposure; the control group remained on the basal diet throughout. The experimental diets were designed as short-term treatment feeds rather than growth-optimization diets. Because fish received the experimental diets for only five days prior to nitrite exposure, small differences in crude protein relative to commercial grow-out formulations were unlikely to affect the hematological parameters evaluated in the study. Both treatments received the same basal formulation, ensuring that dietary composition did not confound treatment comparisons.

### 2.4. Nitrite Exposure Protocol

Acute nitrite intoxication was induced by pumping water from a 1000 L reservoir containing reagent-grade sodium nitrite to maintain target concentrations in each tank. Fish were first exposed to 30 mg/L NO_2_–N for 24 h to cause a sublethal challenge. Because no obvious stress behaviors were observed after 24 h, the concentration was increased to 90 mg/L NO_2_–N for a further 24 h. These doses approximate the 96 h LC_50_ values reported for Nile tilapia [12] and allowed evaluation of MB efficacy under progressively severe conditions.

### 2.5. Sampling and Hematological Analyses

At the end of the 48 h exposure, ten fish per treatment were randomly selected for blood and tissue analyses (*n* = 10). Fish were anesthetized by immersion bath in a solution containing 1.5 mL/L eugenol. Blood was drawn by caudal venipuncture using EDTA-coated syringes. The hemoglobin concentration was measured by the cyanmethemoglobin method with a commercial kit. Hematocrit was determined using microcapillary centrifugation, and plasma protein via portable refractometer. Erythrocyte counts were performed manually in a Neubauer chamber with Natt–Herrick staining, and mean corpuscular volume, mean corpuscular hemoglobin and mean corpuscular hemoglobin concentration were calculated by standard formulas. The sample size was selected to balance statistical sensitivity with ethical considerations regarding animal use. Sampling ten fish per treatment allowed detection of treatment effects on hematological variables while maintaining a modest total number of experimental animals.

### 2.6. Methemoglobin Assay

A methemoglobin quantification assay was specifically designed to address the need for precise measurement of methemoglobin levels due to the unique behavior of fish blood samples. The principle of this assay relied on the light absorbance properties of methemoglobin, which peaks at 633 nm, allowing for a clear distinction from other hemoglobin forms. By adding sodium nitrite to the blood sample, all hemoglobin is converted into methemoglobin, enabling the comparison of pre-treated and induced-MetHb levels within the same sample. A key aspect of the assay is its ability to correct for the minor absorbance of oxyhemoglobin at 633 nm. Without this correction, initial absorbance can be overestimated, leading to inaccurate methemoglobin percentage quantification.

A 50 μL aliquot of whole blood was added to 1250 μL of phosphate-buffered solution (10 mM KH_2_PO_4_, 6 mM Na_2_HPO_4_, pH = 6.6) and vortexed to promote erythrocyte lysis and hemoglobin oxygenation. The resulting homogenate was transferred to Eppendorf tubes and centrifuged at 10,000 RPM at 20 °C for 3 min. After centrifugation, 1000 μL of the supernatant was transferred to a cuvette for the first absorbance reading (A1) at a wavelength of 633 nm using a spectrophotometer (Shimadzu UV-1800, Kyoto, Japan). The cuvette still containing the sample supernatant received 10 μL of 5% sodium nitrite solution and was incubated for 30 min, followed by a second reading (A_2_) at the same wavelength. The background absorbance (A_H_) attributable to Hb was estimated as A_H_ = 11.6 × Hb, which was derived from a calibration assay using hemoglobin standard. The methemoglobin percentage was calculated as (A_1_ − A_H_)/A_2_) × 100. The concentration of circulating methemoglobin was calculated based on the hemoglobin concentration and methemoglobin percentage.

### 2.7. Histological Analyses

Gill filaments were excised from five fish per tank, fixed in 10% buffered formalin, dehydrated through graded ethanol, embedded in paraffin and sectioned at 5 µm. Sections were stained with Harris hematoxylin and eosin and examined under light microscopy. Lesions were classified descriptively because no lesion scoring system was applied.

In addition to histology, gill coloration was visually inspected for each fish at the time of sampling. No quantitative color scores were assigned. To illustrate the most pronounced differences in gill color between treatments, one fish with obvious brownish gills (consistent with methemoglobinemia) from the control group and one fish with vivid red gills from the MB group were photographed. Color analysis of these two representative images was performed in ImageJ software (version 1.54p) using the RGB profile plot function on a 20 × 20-pixel region of the same anatomical area. These color observations were descriptive and not replicated across multiple individuals.

### 2.8. Statistical Analysis

Data were analyzed using R software (version 4.5.3). Prior to hypothesis testing, data were evaluated for normality and homogeneity of variance using the Shapiro–Wilk and Levene tests, respectively. Statistical differences between treatments were determined using either a Student’s *t*-test or a Welch’s *t*-test, depending on the equality of variances, with a significance level of *p* < 0.05. The experimental unit was the fish; values were presented as means ± standard deviation of 10 fish per treatment.

## 3. Results

### 3.1. Survival and Clinical Observations

**Figure 1:**
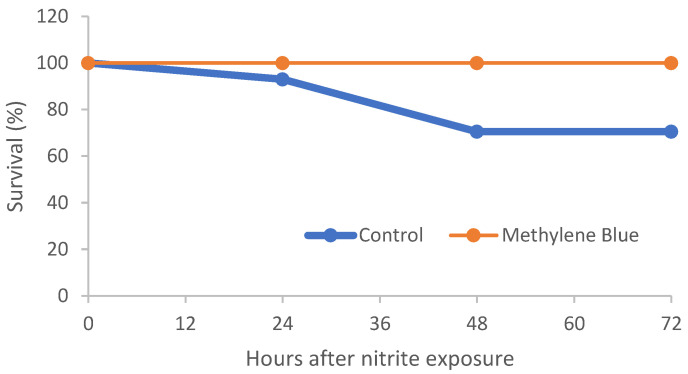
Survival of Nile tilapia fed control or 0.1% methylene blue (MB) diets after five days of feeding and subsequent acute nitrite exposure (30 and 90 mg/L NO_2_–N).

During the initial 24 h at 30 mg/L NO_2_–N, two deaths occurred in the control tanks, whereas no MB-fed fish died or showed abnormal behavior. Upon escalation to 90 mg/L NO_2_–N, control fish displayed erratic swimming, lethargy, and bottom-dwelling behavior (Video S1, Supplementary Material) and suffered three additional mortalities, leaving a final survival of 25/30 fish (≈83%); in contrast, MB-treated fish maintained normal feeding and activity and experienced zero mortalities (Figure 1).

### 3.2. Hematological Responses

The hematological variables are summarized in Table 2. Control fish had significantly higher hemoglobin concentration (5.06 ± 1.04 g/dL vs. 3.95 ± 1.25 g/dL) and erythrocyte counts (1.84 ± 0.82 × 10^6^ mm^−3^ vs. 1.29 ± 0.31 × 10^6^ mm^−3^) compared with MB-treated fish. The methemoglobin percentage (64.85 ± 9.12% vs. 57.97 ± 10.77%) and methemoglobin concentration (3.25 ± 0.67 g/dL vs. 2.22 ± 0.68 g/dL) were both significantly higher in controls. Mean corpuscular volume was lower in control fish (121.83 ± 77.92 fL) than in MB-fed fish (165.71 ± 69.04 fL). No significant differences were detected for hematocrit, plasma protein, mean corpuscular hemoglobin or mean corpuscular hemoglobin concentration.

### 3.3. Gill Histology

**Figure A1:**
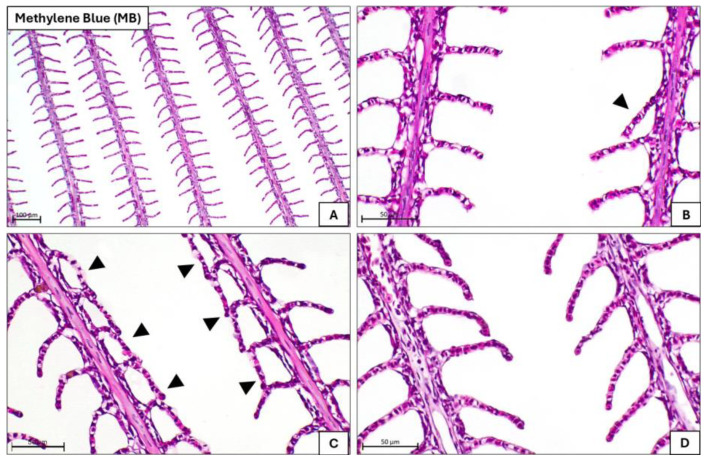
Photomicrograph of the gills from the fish in the group treated with methylene blue (MB). (**A**) Branchial filaments with preserved architecture; (**B**) filaments with preserved architecture, presenting only a tortuous lamella in a focal point (black triangle); (**C**) filaments with preserved architecture, presenting tortuous branchial lamellae in multifocal points (black triangles); and (**D**) filaments with preserved architecture.

**Figure A2:**
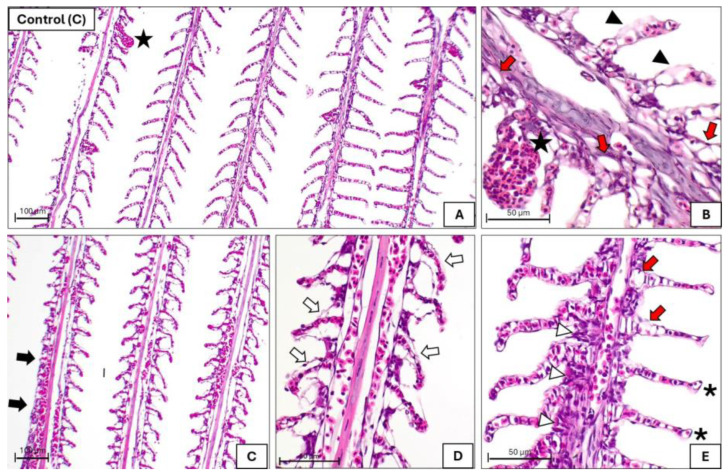
Photomicrograph of the gills from the control group fish (**C**). In all images, it is possible to observe lesions: (**A**) lamellar aneurysm (black star); (**B**) lamellar aneurysm (black star) and lamellar edema (black triangles); capillary congestion (red arrows); (**C**) region of lamellar fusion (black arrows); (**D**) epithelial displacement (white arrows); and (**E**) epithelial thickening, cellular hyperplasia, and mucous cell proliferation (white triangles); capillary congestion (red arrows) and mild hypertrophy (black asterisk).

Gill sections from MB-treated fish displayed largely preserved filament and lamellar architecture with only occasional tortuous lamellae (Figure A1). In contrast, control gills showed multiple lesions, including lamellar aneurysm, edema, capillary congestion, lamellar fusion, epithelial displacement, epithelial thickening, mucous cell proliferation and cellular hyperplasia (Figure A2). These qualitative observations suggest that MB treatment reduced branchial injury during nitrite intoxication; however, lesion scoring was not performed, and differences should be interpreted cautiously.

**Figure A3:**
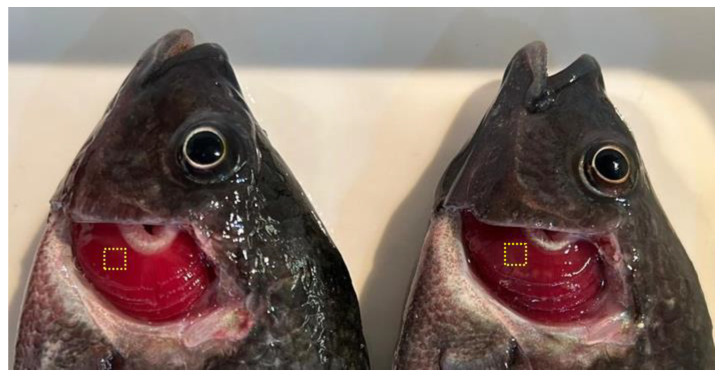
Gill coloration of the fish: on the left, a fish from the methylene blue-treated group; on the right, a fish from the control group. Yellow squares indicate the 20 × 20-pixel area where the RGB profile was sampled.

**Figure A4:**
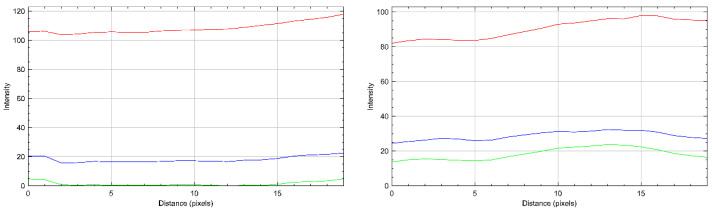
RGB profiles of the sampled area of the gills. (**Left**): A fish from the methylene blue-treated group. The red, green, and blue lines represent the intensity profiles of the corresponding color channels generated from the analyzed gill image area. (**Right**): A fish from the control group.

Gill coloration differed visibly between treatments. Representative photographs showed vivid red gills in the MB-treated fish and brownish gills in the control fish (Figure A3). An exploratory RGB profile analysis of the two representative images indicated a higher proportion of the red channel in the MB gill and relatively elevated green and blue channels in the control gill (Figure A4). Because these analyses were conducted on only one fish per treatment, these color observations are illustrative and should not be generalized without proper caution and replication.

## 4. Discussion

The present study demonstrated that dietary administration of methylene blue can substantially attenuate the physiological consequences of acute nitrite exposure in Nile tilapia. Fish receiving MB-supplemented feed experienced no mortality and maintained normal behavior during a severe nitrite challenge that caused death and clear signs of hypoxic distress in the control group. These outcomes were accompanied by significantly lower circulating methemoglobin levels and reduced erythrocyte responses in MB-treated fish, indicating that MB mitigated the oxidation of hemoglobin and preserved functional oxygen transport. Together, these findings suggest that oral delivery of methylene blue can provide systemic protection against nitrite-induced methemoglobinemia in fish.

It is worth noting that the experimental protocol reported here evaluated a preventive feeding strategy rather than a post-exposure therapeutic treatment. In practice, nitrite spikes in recirculating aquaculture systems often occur following predictable disturbances such as biofilter instability, sudden increases in feeding or system maintenance events. Under such circumstances, preventive management strategies are commonly used in aquaculture. Therefore, administering MB in feed prior to a predicted nitrite challenge allowed evaluation of whether the compound could attenuate methemoglobin formation during exposure. Because methylene blue functions as a catalytic redox mediator rather than a stored antioxidant, its protective effect does not depend on tissue accumulation but on its availability in the bloodstream during periods of oxidative stress. For this reason, future studies should examine whether MB administered after the onset of intoxication can reverse established methemoglobinemia.

From an operational perspective, MB-medicated feed should be viewed as an emergency tool for situations where acute nitrite accumulation is imminent or ongoing and rapid, scalable intervention is required. In such cases, administering MB through routine feeding may help maintain functional oxygen transport during the crisis while standard management measures are implemented (e.g., chloride addition to reduce nitrite uptake, temporary feed restriction, and partial water exchange where feasible). Importantly, the present study did not address post-exposure “rescue” dosing; therefore, future work should evaluate whether initiating MB feeding only after clinical signs appear can reverse established methemoglobinemia.

The present results showed that incorporating a modest amount of MB (0.1%) into feed eliminated mortality and prevented visible distress during a severe nitrite challenge. These findings suggest that maintaining a circulating redox-active compound in the bloodstream through routine feeding may physiologically buffer fish against acute hemoglobin oxidation during nitrite spikes. Oral delivery avoids handling stress and minimizes the volume of chemicals introduced into the system water.

The control group had higher Hb and erythrocyte counts than the MB-fed group, despite also showing higher methemoglobin burden. Elevated Hb and erythrocytes are characteristic of acute hypoxia responses in fish and likely reflect splenic release of stored erythrocytes and accelerated erythropoiesis in an attempt to maintain oxygen delivery [13,14]. Thus, the greater Hb in control fish should not be interpreted as improved oxygenation but rather as a compensatory reaction to more severe hypoxic stress. In contrast, MB-fed fish showed lower methemoglobin percentage and concentration, indicating that MB attenuated the oxidation of Hb and reduced the hypoxic challenge. Consequently, MB-fed fish did not mount the same erythropoietic response and maintained functional oxygen transport without clinical signs. The reduction in circulating methemoglobin in MB-treated fish is consistent with the known redox activity of methylene blue, which can function as an electron carrier facilitating the enzymatic reduction of ferric iron (Fe^3+^) in methemoglobin back to the functional ferrous state (Fe^2+^) in hemoglobin. This mechanism explains the lower hypoxic stress response observed in MB-treated fish. The lower mean corpuscular volume observed in control fish may also represent the presence of smaller, immature erythrocytes released during the acute-stress response. Because hematological measurements were taken at a single end point and only surviving fish were sampled, these results could underestimate the severity of hemolysis or oxidative damage in fish that succumbed earlier. Future studies should incorporate serial sampling to examine temporal changes.

Gill lesions observed in control fish were typical of nitrite or other pollutant substances, including lamellar aneurysm, edema, fusion and epithelial hyperplasia. Such remodeling of gill architecture can reduce contaminant uptake but also diminishes respiratory surface area and increases diffusive distance, thereby exacerbating hypoxia. MB-treated fish lacked these lesions, suggesting that MB mitigated tissue damage during nitrite exposure. In addition to structural lesions, we noted clear differences in gill color between treatments: MB-fed fish had bright red gills, whereas control fish showed brownish coloration consistent with methemoglobinemia. An exploratory RGB profile analysis of two representative images supported this qualitative observation, showing higher relative red intensity in the MB sample and elevated green and blue components in the control sample. Because color assessments were not scored and were based on only one of the most representative fish of each treatment, these observations are mostly illustrative and require replication. Overall, preserved branchial structure and normal coloration are consistent with the absence of behavioral distress and the improved oxygen transport profile in MB-fed fish. Because lesion assessment and color analysis were qualitative, the observed differences should be confirmed in future studies using blinded scoring, morphometric measurements and replicated color metrics.

Several caveats temper the prompt application of these findings. First, only a single MB dose was tested; dose–response trials are necessary to determine the minimum effective and maximum safe inclusion. Second, MB feeding began before nitrite exposure, so the protocol assessed prevention/attenuation rather than post-intoxication rescue. Future work should evaluate whether MB delivered only after nitrite exposure can reverse established methemoglobinemia. Third, the experiment considered the fish as experimental units, limiting statistical power and preventing robust analysis of tank effects. Fourth, gill histology was descriptive; quantitative scoring of lesion prevalence and severity would strengthen conclusions. Finally, MB residues were not analyzed; regulatory assessments of tissue residues and consumer safety are prerequisite for practical adoption. Methylene blue is classified differently across jurisdictions and is not currently approved for use in food fish in several regions. The present study does not advocate immediate commercial application but rather evaluates the physiological efficacy of a feed-based delivery strategy. Before practical adoption could be considered, additional studies will be required to determine tissue residue kinetics, withdrawal periods and regulatory compliance.

Ultimately, it should also be recognized that methylene blue may produce hematological or oxidative effects at elevated doses or prolonged exposure, as reported in other fish species. Consequently, determining the minimum effective dietary inclusion and evaluating potential toxicological thresholds represent important priorities for future research.

## 5. Conclusions

Feeding Nile tilapia a diet containing 0.1% methylene blue for five days prior to an acute nitrite challenge improved survival, reduced methemoglobin burden and mitigated gill injury compared with fish receiving a basal diet. MB-treated fish showed no mortality and maintained normal behavior under high nitrite concentrations that caused distress and death in controls. Hematological data suggested that MB attenuated the oxidative conversion of hemoglobin to methemoglobin, thereby preserving functional oxygen transport and reducing the need for compensatory erythropoiesis.

These results provide preliminary evidence that methylene blue delivered orally through feed may help buffer fish against nitrite-induced methemoglobinemia during acute water quality crises. However, the present findings should be interpreted within the scope of a controlled experimental study. Further research is required to determine optimal dosing, evaluate post-exposure therapeutic efficacy, quantify tissue residues, and assess regulatory considerations before practical application in aquaculture systems.

## Figures and Tables

**Table 1:** Ingredient composition of the basal feed.

Ingredients	Inclusion (%)
Soybean meal	50.00
Corn	39.7
Soybean oil	2.60
DL-methionine	0.10
Vitamin-mineral mix	1.00
Dicalcium phosphate	4.75
Calcitic limestone	1.85

**Table 2:** Hematological parameters of Nile tilapia receiving control or MB-medicated feed and exposed to acute nitrite intoxication (means ± SD, *n* = 10 fish per treatment).

Parameter	MB Feed	Control	*p*-Value
Hemoglobin (g/dL)	3.95 ± 1.25	5.06 ± 1.04	0.009
Methemoglobin (%)	57.97 ± 10.77	64.85 ± 9.12	0.046
Circulating MetHb (g/dL)	2.22 ± 0.68	3.25 ± 0.67	<0.001
Erythrocytes (×10^6^/mm^3^)	1.29 ± 0.31	1.84 ± 0.82	0.017
Hematocrit (%)	20.05 ± 5.03	19.83 ± 4.62	0.894
Plasma protein (g/dL)	4.37 ± 0.50	4.61 ± 0.53	0.204
MCV (fL) ^1^	165.71 ± 69.04	121.83 ± 77.92	0.041
MCH (pg) ^2^	32.62 ± 14.90	31.32 ± 15.91	0.783
MCHC (%) ^3^	20.63 ± 2.09	27.51 ± 3.39	0.063

^1^ Mean corpuscular volume. ^2^ Mean corpuscular hemoglobin. ^3^ Mean corpuscular hemoglobin concentration.

## Data Availability

The data presented in this study are openly available in Mendeley Data at https://doi.org/10.17632/yxgzb8rxw4.1.

## Supplementary Materials

The following supporting information can be downloaded at https://www.mdpi.com/article/10.3390/ani16071042/s1, Video S1: mb-video.mp4.

## Abbreviation

The following abbreviation were used in this manuscript:

MB	Methylene Blue

## References

[1] Zhang T.T., Ma P., Yin X.Y., Yang D.Y., Li D.P., Tang R. (2022). Acute Nitrite Exposure Induces Dysfunction and Oxidative Damage in Grass Carp Isolated Hemocytes. J. Aquat. Anim. Health.

[2] Kocour Kroupová H., Valentová O., Svobodová Z., Šauer P., Máchová J. (2018). Toxic Effects of Nitrite on Freshwater Organisms: A Review. Rev. Aquac..

[3] Chatzinikolaou P.N., Margaritelis N.V., Paschalis V., Theodorou A.A., Vrabas I.S., Kyparos A., D’Alessandro A., Nikolaidis M.G. (2024). Erythrocyte Metabolism. Acta Physiol..

[4] Shen C., Cao S., Mohsen M., Li X.S., Wang L., Lu K.L., Zhang C.X., Song K. (2024). Effects of Chronic Nitrite Exposure on Hematological Parameters, Oxidative Stress and Apoptosis in Spotted Seabass (*Lateolabrax maculatus*) Reared at High Temperature. Aquac. Rep..

[5] Banerjee P., Garai P., Saha N.C., Saha S., Sharma P., Maiti A.K. (2023). A Critical Review on the Effect of Nitrate Pollution in Aquatic Invertebrates and Fish. Water Air Soil Pollut..

[6] Díaz V., Ibáñez R., Gómez P., Urtiaga A.M., Ortiz I. (2012). Kinetics of Nitrogen Compounds in a Commercial Marine Recirculating Aquaculture System. Aquac. Eng..

[7] Buzga M., MacHytka E., Dvoracková E., Švagera Z., Stejskal D., Máca J., Král J. (2022). Methylene Blue: A Controversial Diagnostic Acid and Medication?. Toxicol. Res..

[8] Lieke T., Meinelt T., Hoseinifar S.H., Pan B., Straus D.L., Steinberg C.E.W. (2020). Sustainable Aquaculture Requires Environmental-Friendly Treatment Strategies for Fish Diseases. Rev. Aquac..

[9] Zhang X., Hui Y., Fang C., Wang Y., Han F., Lou X., Fodjo E.K., Cai Y., Kong C. (2021). Determination of Methylene Blue and Its Metabolite Residues in Aquatic Products by High-Performance Liquid Chromatography–Tandem Mass Spectrometry. Molecules.

[10] Bortz B.M. (1977). The Administration of Tetramethylthionine Chloride as a Treatment for Nitrite-Induced Methemoglobinemia in Rainbow Trout (Salmo Gairdneri).

[11] Wedemeyer G.A., Yasutake W.T. (2011). Prevention and Treatment of Nitrite Toxicity in Juvenile Steelhead Trout (*Salmo gairdneri*). J. Fish. Board Can..

[12] Atwood H.L., Fontenot Q.C., Tomasso J.R., Isely J.J. (2001). Toxicity of Nitrite to Nile Tilapia: Effect of Fish Size and Environmental Chloride; Toxicity of Nitrite to Nile Tilapia: Effect of Fish Size and Environmental Chloride. N. Am. J. Aquac..

[13] Wu C.B., Zheng G.D., Zhao X.Y., Zhou S., Zou S.M. (2020). Hypoxia Tolerance in a Selectively Bred F4 Population of Blunt Snout Bream (*Megalobrama amblycephala*) under Hypoxic Stress. Aquaculture.

[14] Abdel-Tawwab M., Monier M.N., Hoseinifar S.H., Faggio C. (2019). Fish Response to Hypoxia Stress: Growth, Physiological, and Immunological Biomarkers. Fish Physiol. Biochem..

